# Segmentation of abdomen MR images using kernel graph cuts with shape priors

**DOI:** 10.1186/1475-925X-12-124

**Published:** 2013-12-03

**Authors:** Qing Luo, Wenjian Qin, Tiexiang Wen, Jia Gu, Nikolas Gaio, Shifu Chen, Ling Li, Yaoqin Xie

**Affiliations:** 1 The Shenzhen Key Laboratory for Low-cost Healthcare, Shenzhen Institutes of Advanced Technology, Chinese Academy of Sciences, Shenzhen, P. R. China; 2 Faculty of Computer, Guangdong University of Technology, Guangzhou, China; 3 University of Chinese Academy of Sciences, Beijing, China

## Abstract

**Background:**

Abdominal organs segmentation of magnetic resonance (MR) images is an important but challenging task in medical image processing. Especially for abdominal tissues or organs, such as liver and kidney, MR imaging is a very difficult task due to the fact that MR images are affected by intensity inhomogeneity, weak boundary, noise and the presence of similar objects close to each other.

**Method:**

In this study, a novel method for tissue or organ segmentation in abdomen MR imaging is proposed; this method combines kernel graph cuts (KGC) with shape priors. First, the region growing algorithm and morphology operations are used to obtain the initial contour. Second, shape priors are obtained by training the shape templates, which were collected from different human subjects with kernel principle component analysis (KPCA) after the registration between all the shape templates and the initial contour. Finally, a new model is constructed by integrating the shape priors into the kernel graph cuts energy function. The entire process aims to obtain an accurate image segmentation.

**Results:**

The proposed segmentation method has been applied to abdominal organs MR images. The results showed that a satisfying segmentation without boundary leakage and segmentation incorrect can be obtained also in presence of similar tissues. Quantitative experiments were conducted for comparing the proposed segmentation with other three methods: DRLSE, initial erosion contour and KGC without shape priors. The comparison is based on two quantitative performance measurements: the probabilistic rand index (PRI) and the variation of information (VoI). The proposed method has the highest PRI value (0.9912, 0.9983 and 0.9980 for liver, right kidney and left kidney respectively) and the lowest VoI values (1.6193, 0.3205 and 0.3217 for liver, right kidney and left kidney respectively).

**Conclusion:**

The proposed method can overcome boundary leakage. Moreover it can segment liver and kidneys in abdominal MR images without segmentation errors due to the presence of similar tissues. The shape priors based on KPCA was integrated into fully automatic graph cuts algorithm (KGC) to make the segmentation algorithm become more robust and accurate. Furthermore, if a shelter is placed onto the target boundary, the proposed method can still obtain satisfying segmentation results.

## Introduction

The recent development of open magnetic resonance imaging has provided new opportunities for next generation image-guided surgical and interventional applications. Image-guided surgery is a standard surgical procedure for abdominal disorders that can reduce surgical trauma and open surgery burdens [1]. However, during surgical planning and surgical navigation based on MR images, there are two problems have to be faced: the shape deformation of the organs and the similarity among abdominal organs. For these reasons, an effective and robust algorithm for abdominal organs segmentation is helpful and very important in image-guided surgery and surgical navigation system [2,3].

Usually, basic image information, such as intensity and gradient, are used for segmentation; Ostu, k-means clustering, region growing, etc. are the most widely used algorithms for segmentation. However, they are not suitable for MR segmentation. Because of its weak boundary, intensity inhomogeneity and noise, the segmentation of MR images is considered a complex procedure [4]. For this reason, developing different advanced and intelligent algorithms for MR image segmentation has become a research hotspot over the last few years.

Abdomen MR image segmentation is a challenging task, because majority of tissues in abdomen are soft tissues, the intensities of abdominal tissues are very similar and shape change in a complex way due to respiratory movements [5]. To provide more information about the tissues and organs in abdomen to the doctors, more effective and robust algorithm to segment abdomen MR images are developed. For example, Hassan et al. [6] proposed a novel method to segment liver MR images automatically. This algorithm utilizes artificial neural networks and watershed algorithm. Moreover, Sheng et al. [7] applied a wavelet-based k-means clustering method to segment the human kidney from MRI data set.

Since the level set method can be performed on a fix Cartesian grid with no need to parameterize these objects, in the past decade it was used more and more frequently in image segmentation [8-11]. Moreover, the level set method can represent contours with complex topology and change their topology in a natural way. However, the algorithms converge to a local minimum easily, so the results are easily affected by the initial values. Additionally, the execution time may be very long in some applications, especially with large images and multi-object segmentations [12,13].

Graph cuts techniques have received considerable attention for their global energy optimal advantages. It is used in more and more image segmentation applications for different medical images, such as MR images [14]. However, it requires to choose the object and background seeds interactively, implying a time-consuming procedure. Kernel graph cuts is a fully automatic algorithm based on graph cuts proposed by Salah et al. [15]. It consists of a multi-region image segmentation algorithm based on graph cuts via kernel mapping of the image data. This algorithm is not successful for abdominal organs segmentation due to the weak boundary and surrounding objects with similar intensities. The graph cuts based on active contours method was proposed by Xu et al. [16]. The method identifies an initial contour around the target and then forms the narrow banded area containing the target via dilation and erosion. The segmentation focuses on image data in the narrow banded area, and separates the abdominal organs with similar intensities. However it cannot solve the weak boundary problem that leads to edge leakage. Integrating the prior information into the segmentation is a popular solution for the weak boundary; the prior information can lead to a more accurate segmentation result according to [17-20]. Asem et al. [21] proposed graphs cuts integrated shape priors, and applied this method to kidney segmentation in abdomen MR images. The result of the segmentation shows that it can overcome the edge leakage, however, it uses the probabilistic model, which increases its complexity; moreover, it also needs interactive operations. Chen et al. [21] integrate shape information generated from Active Appearance Model (AAM) into graph cuts for abdominal 3D organ segmentation. However, the long computation time and the dependence of initial location limit its applications. Comparing to the linear PCA (principle component analysis), the nonlinear PCA model performs better on problems with nonlinear deformation [22]. Malcolm et al. [23] proposed a segmentation model that integrates the KPCA-based shape priors into graph cuts, but the model executes iteratively so it is time-consuming.

In this paper, we propose a new method, which combines kernel graph cuts with KPCA to segment abdominal organs. First, a seed point is chosen inside the target organ, and then the region growing algorithm is used to obtain approximately segmentation. Second, image morphology operations (dilation and erosion) are used to form an initial contour close to the target organ. Third, KPCA is used to obtain the shape priors by training the relevant shape templates set. At last, the shape priors are integrated into kernel graph cuts to make a better segmentation. The main contribution of this paper is the proposal of novel methods that combine kernel graph cuts algorithm with shape priors for abdominal organs extraction. The shape priors based on KPCA help our algorithm to increase segmentation accuracy while intensity is not sufficient to obtain accurate segmentation results.

This paper is organized as follows. Section Methodology describes the methodology which gives a description of kernel graph cuts and shape priors based on KPCA and detailed introduction of our proposed method. Section Experiments presents the experiments results obtained using the proposed novel method. Finally, the discussion about the algorithms is presented and conclusions are drawn in Section Discussion and Section Conclusions, respectively.

## Methodology

This section starts by briefly describing the KGC and KPCA.

### Kernel graph cuts

Graph cuts algorithm was introduced by Boykov et al. [14] for binary image segmentation application. The purpose is to segment an object from a given image using a set of seeds (object and background) placed by user.

The graph cuts algorithm aims to cast the energy-based image segmentation problem into a graph structure global min-cut problem. The energy function of graph cuts contains two terms: a region-based term *R(A)*and a boundary term *B(A)*, where *A* stands for an object or background pixel assignment. The region-based term evaluates the penalty for assigning a particular pixel to a given region. The boundary term evaluates the penalty for assigning two neighboring pixels to different regions. These two terms often weight by 0 ≤ *ϵ* ≤ 1 for relative influence, and the energy function is expressed as follows:

(1) $$E\left(A\right)=\epsilon \cdot R\left(A\right)+\left(1-\epsilon \right)\cdot B\left(A\right)$$

KGC was proposed by Salah et al. [15] for automatic segmentation by mapping image data into high dimension through kernel function. Graph cuts method is a supervised algorithm which requires user intervention for choosing seeds (object & background). The proposed energy function contains two terms: an original kernel-induced data term which evaluates the deviation of the mapped image data and a regularization term expressed as a function of the region indices. The energy function is expressed as follows:

(2) $$E\left(\left\{\mu_{l}\right\},\delta \right)={\sum_{l\in L}\sum_{p\in R_{l}}\left(\phi \left(\mu_{l}\right)-\phi \left(I_{p}\right)\right)}^{2}+\alpha \sum_{\left\{p,q\right\}\in D}r\left(\delta \left(p\right),\delta \left(q\right)\right)$$

Where *E*({μ_1_},*δ*) measures kernel-induced non Euclidean distances between the observations and the regions parameters *μ*_*1*_. *φ* is a nonlinear mapping from the observation space *I* to a higher dimensional mapped space *J*, and the radial basis function (gauss function) kernel is used as usual. *a* is a positive factor. *δ* is an indexing function which assigns each point of the image to a region. *l* ∈ *L* Is a pixel label in some finite set of labels *L. P∈R*_*1*_ is a pixel in each region which is characterized by one label *l*. R(*δ((p),δ(q,*)) is a smooth regularization function and *D* is a neighborhood set containing all pairs of neighboring pixels *{p,q}*.

According to the Mercer’s theorem [24], which states that kernel function can be expressed as a dot product in a high-dimensional space, explicitly the mapping *φ* is not available. Instead, the kernel function is as follow:

(3) $$K\left(y,z\right)=\phi {\left(y\right)}^{T}\cdot \phi \left(z\right)\;,\;\forall \left(y,z\right)\in I^{2}$$

Substitution of the kernel function gives:

(4) $$\begin{array}{ll} \;J_{K}\left(I_{p},\mu \right) & ={∥\phi \left(I_{p}\right)-\phi \left(\mu \right)∥}^{2}\\ & ={\left(\phi \left(I_{p}\right)-\phi \left(\mu \right)\right)}^{T}\cdot \left(\phi \left(I_{p}\right)-\phi \left(\mu \right)\right)\\ & =\phi {\left(I_{p}\right)}^{T}\phi \left(I_{p}\right)-\phi {\left(\mu \right)}^{T}\phi \left(I_{p}\right)-\phi {\left(I_{p}\right)}^{T}\phi \left(\mu \right)+\phi {\left(\mu \right)}^{T}\phi \left(\mu \right)\\ & =K\left(I_{p},I_{p}\right)+K\left(\mu ,\mu \right)-2K\left(I_{p},\mu \right)\;,\;\mu \in \left\{\mu_{l}\right\} \end{array}$$

Equation (4) is solved for∥*ϕ*(*I*_*p*_) − *ϕ*(*μ*)∥^2^ and substituted in (2). Thus, the kernel-induced energy function is given by:

(5) $$E\left(\left\{\mu_{l}\right\},\delta \right)=\sum_{l\in L}\sum_{p\in R_{l}}J_{K}\left(I_{p},\mu_{l}\right)+\alpha \sum_{\left\{p,q\right\}\in D}r\left(\delta \left(p\right),\delta \left(q\right)\right)$$

Now, based on the kernel-induced energy function, the graph cuts algorithm can be executed to segment images more efficiently.

### Kernel principle component analysis

KPCA is a nonlinear feature extractor performed in the feature space *F*. The basic idea of the method is to map the data from the input space *S* to a feature space *F* via nonlinear map *ϕ*:*S*→*F*. Because KPCA is able to capture nonlinear features in the data comparing to linear PCA, it can be used more effectively if a pre-image of the projection in the feature space is available. Rathi et al. [22] proposed a novel method to reconstruct a unique approximate pre-image of a feature vector and applied it for statistical shape analysis.

To form the statistical model of shape space *S*, the pre-image of the projection (in the KPCA space) of a test point *x∈S* should be found, as shown in Figure 1 from [25]. Let {*x*_*1*_*, X*_*n*_}⊂*S* be a set of aligned training shapes represented by binary mask where 1 is object and 0 is background and spread as vectors.

**Figure 1:**
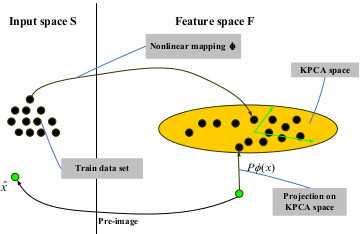
The pre-image problem in KPCA.

First, the *N*×*N* kernel matrix *K* with the radial basis function (gauss function) has to be computed:

(6) $$K_{i\;j}=k\left(x_{i},x_{j}\right)=\exp\left(\frac{-{∥x_{i}-x_{j}∥}^{2}}{2\sigma^{2}}\right)$$

Second, following Eigen decomposition has to be considered :

(7) $$\mathrm{HKH}=U\sum U^{T}$$

*H* is the centering matrix given by $H=C-\frac{1}{N}cc^{T}$,*C* is the *N*×*N* identity matrix, *c* = [11 … 1]^*T*^ is an *N*×1 vector, *U* = [∂_1_, ⋯ ∂_*N*_]^*T*^ with ∂_*i*_ = [*a*_*i*1_, …, *a*_*iN*_]^*T*^ is the matrix containing the eigenvectors and Σ = *diag*(*λ*_1_, …, *λ*_*N*_) contains the corresponding eigen values.

Third, given a point *x∈S* one can compute its projection *Pϕ(x)∈F* and a subspace is spanned by the first n eigenvectors given by:

(8) $$\mathrm{P\varphi}\left(x\right)=\sum_{k=1}^{n}\beta_{k}V_{k}+\bar{\varphi }$$

Where $\bar{\varphi }=\frac{1}{N}\sum_{i=1}^{N}\varphi \left(x_{i}\right)$; $\tilde{\varphi }$ is the map centralized by $\tilde{\varphi }\left(x\right)=\varphi \left(x\right)-\bar{\varphi }$. $V_{k}=\sum_{i=1}^{N}\frac{a_{k\;i}}{\sqrt{\lambda_{k}}}\tilde{\varphi }\left(x_{i}\right)$ is the *k*th orthogonal eigenvector of the covariance matrix in *F*. The projection of test point *x* in *F* project onto the *k*th component by *B*_*k*_. Then $\beta_{k}=\frac{1}{\sqrt{\lambda_{k}}}\sum_{i=1}^{N}a_{k\;i}\tilde{k}\left(x,x_{i}\right)$

$$\mathrm{where}\;\tilde{k}\left(x,y\right)=<\tilde{\varphi }\left(x\right),\tilde{\varphi }\left(y\right)>=k\left(x,y\right)-\frac{1}{N}c^{T}k_{x}-\frac{1}{N}c^{T}k_{y}+\frac{1}{N^{2}}c^{T}\mathrm{Kc}$$

$$\mathrm{with}\;k_{x}={\left[k\left(x,x_{1}\right),\ldots ,k\left(x,x_{N}\right)\right]}^{T}$$

Finally, the method in [22] is used to compute the approximate pre-image $\hat{x}$:

(9) $$\hat{x}=\frac{\sum_{i=1}^{N}{\tilde{\gamma }}_{i}\left(\frac{1}{2}\left(2-{∥\varphi \left(x_{i}\right)-\mathrm{P\varphi}\left(x\right)∥}^{2}\right)\right)x_{i}}{\sum_{i=1}^{N}{\tilde{\gamma }}_{i}\left(\frac{1}{2}\left(2-{∥\varphi \left(x_{i}\right)-\mathrm{P\varphi}\left(x\right)∥}^{2}\right)\right)}$$

And the distance used in feature space in equation (9) is defined as follows:

(10) $${∥\varphi \left(x_{i}\right)-\mathrm{P\varphi}\left(x\right)∥}^{2}={\left({\tilde{k}}_{x}+2H\left(\frac{1}{N}K\;c-k_{x\;i}\right)\right)}^{T}M{\tilde{k}}_{x}+\frac{1}{N^{2}}c^{T}K\;c+K_{i\;i}-\frac{2}{N}c^{T}k_{x\;i}$$

where $M=\sum_{i=1}^{N}\frac{1}{\lambda_{i}}\partial_{k}{\partial_{k}}^{T}$, *γ* = [∂_1_, …, ∂_*n*_]*β*, $\tilde{\gamma }=\gamma +\frac{1}{N}\left(1-c^{T}\gamma \right)$.

The pre-image $\hat{x}$ contains the information that is used to form the shape priors. In following section, the proposed method is presented and summarized.

### Proposed method

**Figure 2:**
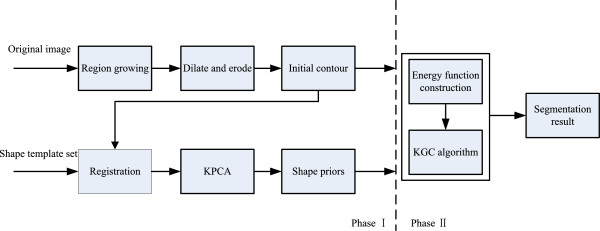
The proposed segmentation framework.

To mitigate the effect of weak boundary in MR images and to segment abdominal organs from surrounding objects with similar intensities, a novel segmentation method is proposed. The method incorporates KPCA with KGC enlightened by [23]. KPCA is used to form the shape priors based on the statistical model proposed by Rathi et al. [22]. Enlightened by the GCBAC (graph cuts based Active Contours) proposed by Xu et al. [16], the initial dilation and erosion contour idea is introduced into the method to locate the position of shape priors. The framework of the segmentation method is shown in Figure 2.

The proposed segmentation framework can be described in two phases.

Phase I is the pre-segmentation phase. It includes two procedures to form the image data.

First, to form the initial contour, a seed point is chosen inside the target region manually, and then the region growing algorithm is used to segment the target region approximately. Based on the result of region growing algorithm, a lot of isolated small regions in the target region are not segmented correctly. For this reason, the morphological dilate and erode operation are executed over the pre-segmented contour using the region growing algorithms. The dilate operation aims to eliminate those isolated small regions and to form a continuous contour around the target region; the erode operation aims to draw the contour near the real target region after the dilate operation. Same morphological structuring element is used for the operation of erode and dilate. As a result, the initial contour is obtained. However, because of the weak boundary in the MR images, a further processing is needed to overcome this problem to reach a more accurate segmentation.

Second step includes obtaining the shape priors. Because of the differences varying from person to person, and different parameters of MR imaging, the shapes of abdominal organs to be segmented differ from each other. Moreover, the respiratory movements make the deformation more complex. Since the deformation is nonlinear, the KPCA is used to train the shape templates determined by experts. Before training the data set, all the shape templates and the initial contour should be aligned. So the image registration is needed here, and only translating, scaling and rotating transforms are taken into consideration during the registration process. Suppose that *X*_*i*_ is the vector of one shape template, one of the shape templates *Xj* is to be chosen, the fittest parameters can be obtained through translating *tj*, scaling and rotating transform *M(s*_*j*_*,θ*_*j*_*)*. The target function is obtained by minimizing the error measure *Ej*:

(11) $$E_{j}={\left(X_{i}-M\left(s_{j},\theta_{j}\right)\left[X_{j}\right]-t_{j}\right)}^{T}W\left(X_{i}-M\left(s_{j},\theta_{j}\right)\left[X_{j}\right]-t_{j}\right)$$

*W* is the weight matrix, and the least square method is usually used to solve it.

Commonly, most applications, such as feature extraction and pattern classification, only need the new features generated by KPCA. However, for some other applications, reconstructing the pre-image from the KPCA features is needed. In this case KPCA feature is not necessary to describe the deformation patterns; on the contrary, it is required to reconstruct the shapes from the KPCA features. For a Gaussian kernel, the pre-image $\hat{x}$ can be obtained using Equations (6-10).

Phase II is the segmentation phase. Since the registration is composed of translating, scaling and rotating transform, and the abdominal organs change from patient to patient, the result of registration contour does not represent the real boundary of MR images, and a more accurate segmentation procedure need to be followed. The image data constructs the graph using energy function (5) in KGC, introducing the shape priors $\hat{x}$ into the data term to overcome the weak boundary. This work aims to use the data term for representing the penalty of pixel attribute to the object or background. Thus, it is assumed that non-uniform shape priors *Pp*(*O*) and P_p_(B) represent penalty of the shape priors attribute to the object or background at a pixel *P*. A parameter *η*(0≤*η*≤1) is also introduced to represent the weight of relative influence between kernel-induced data term *J*_*K*_ and shape priors, so the new data terms can be written as follows:

(12) $$R_{p}\left(O\right)=\eta \cdot J_{K}\left(I_{p},\mu_{O}\right)+\left(1-\eta \right)\cdot P_{p}\left(O\right)$$

(13) $$R_{p}\left(B\right)=\eta \cdot J_{K}\left(I_{p},\mu_{B}\right)+\left(1-\eta \right)\cdot P_{p}\left(B\right)$$

Since the pre-image has value between 0 and 1, *P*_*p*_*(O)* is directly used to represent $\hat{x}$ and set *Pp = (***1-***P*_*p*_*(O))*. The smooth term used the original term. At last, based on Equation (5), the new energy function is given by:

(14) $$E\left(\left\{\mu_{l}\right\},\delta \right)=\sum_{l\in L}\sum_{p\in R_{l}}\left(\eta \cdot J_{K}\left(I_{p},\mu_{l}\right)+\left(1-\eta \right)\cdot R_{p}\left(l\right)\right)+\alpha \sum_{\left\{p,q\right\}\in D}r\left(\delta \left(p\right),\delta \left(q\right)\right)$$

Where *l* must be *O* or *B*, which stands for object or background. Thus, the multi-region segmentation of KGC turns to be the binary segmentation of object or background. The new energy function is used to construct graph, compute min-cut and get the segmentation.

## Experiments

Segmentation is performed on abdominal organs in the abdomen MR images of T1 sequence, and the proposed novel segmentation method is validated using the MATLAB 7.11 program on Windows XP with Lenovo PC with Intel (R) Core (TM) 2 Duo CPU, E7500, and tested using the liver and kidney dataset in abdomen MR images with size of 462 × 310 pixels. All MR images are obtained by Siemens 3.0T MR imaging equipment and all the shape templates are segmented manually by different experts. The size of train set is 30.

### Validation

**Figure 3:**
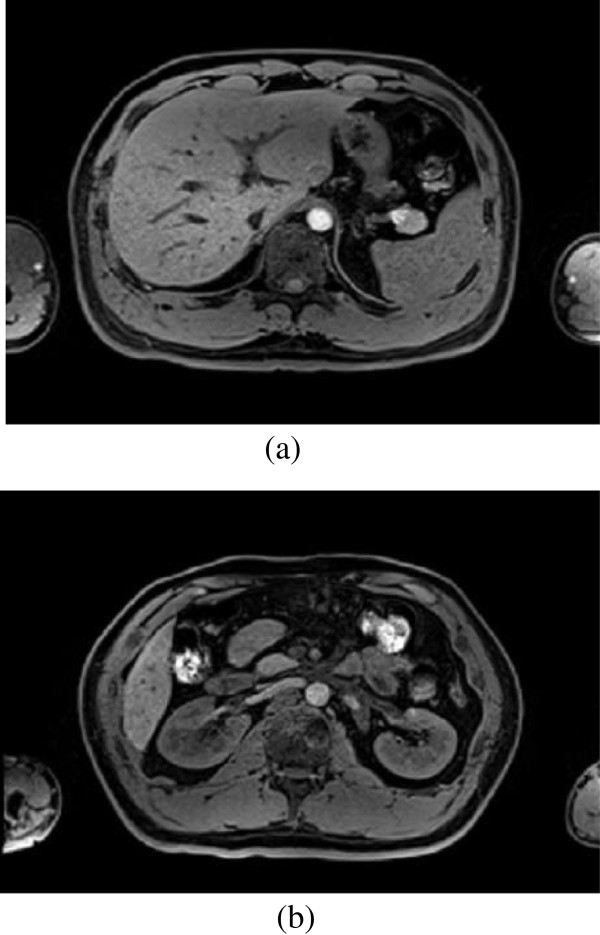
Original image, (a)the abdomen MR image of liver; (b)the abdomen MR image of kidney.

First, the original MR images have to be segmented as shown in Figure 3. Figure 3 (a) is used to segment liver in abdominal organs, and Figure 3 (b) is used to segment both of the two kidneys in abdominal organs.

**Figure 4:**
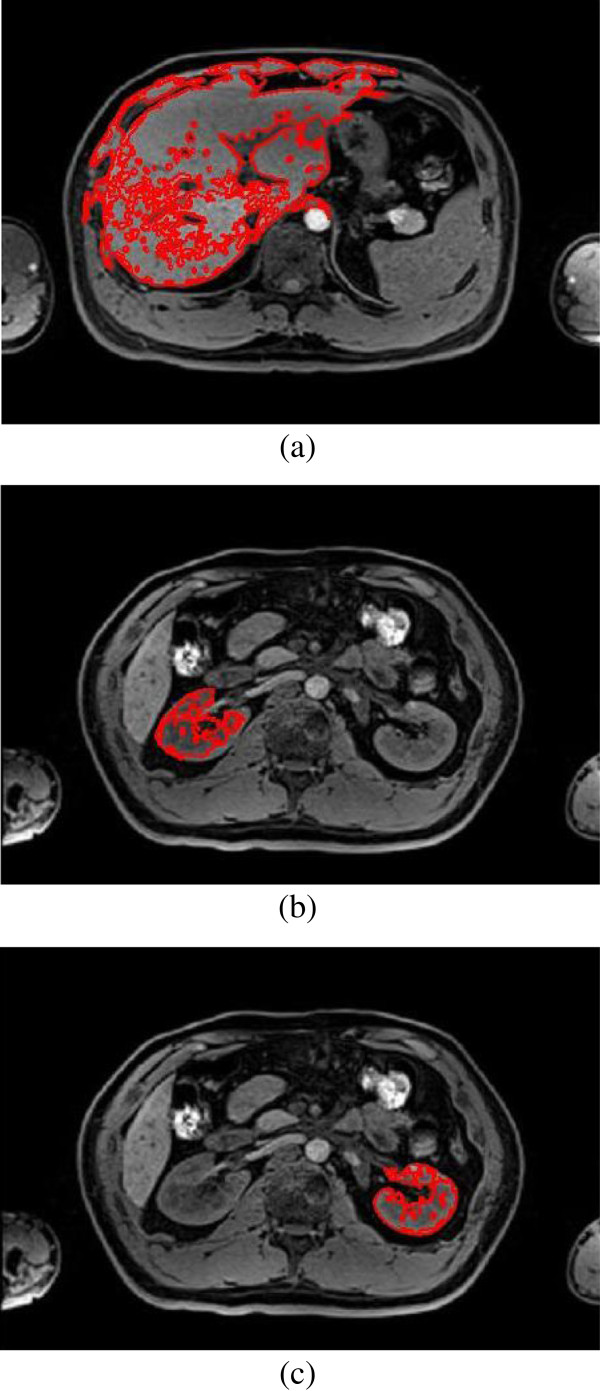
Region growing algorithm, (a) liver; (b) right kidney; (c) left kidney.

Second, a seed point is placed inside the liver and kidney region in the MR images, and then the region growing algorithm is used to segment the image approximately. The result of region growing algorithm is shown in Figure 4. From the Figure 4, it can be seen that many isolated small regions are not segmented, and boundary leakage and incorrect segmentation are observed. Many blood vessels exist inside the organ tissues and the noise caused by MR equipment lead to lots of isolated small regions; meanwhile the soft tissues are very similar and the overlaps between different soft tissues lead to weak boundary. So, more work is needed to solve this problem.

**Figure 5:**
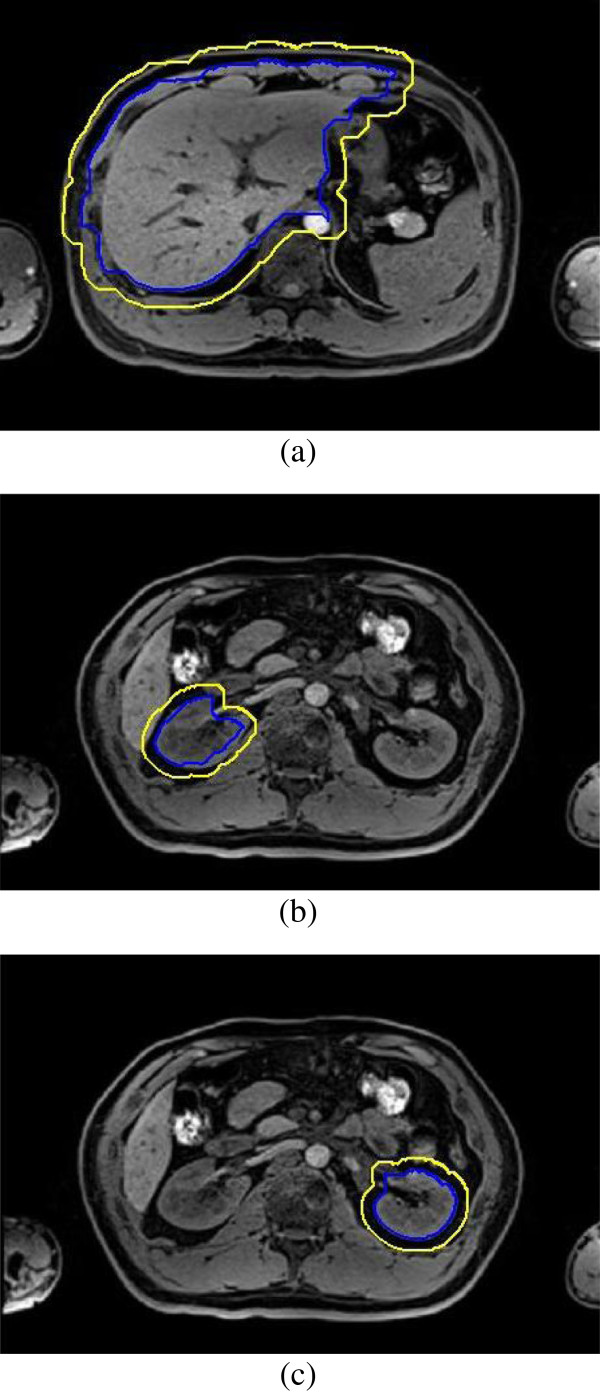
Dilation (yellow contour) and erosion (blue contour), (a) liver(radius = 15); (b) right kidney(radius = 10); (c) left kidney(radius = 10).

Third, the morphological dilate and erode operation are implemented to fix the problem caused by region growing algorithm. The result contour of region growing is dilated and then eroded as demonstrated in Figure 5, the yellow contour is the result of dilation and the blue contour is the result of erosion. The type of morphological structuring element that is used in dilation and erosion is a disk whose radius can be adjusted depending on the result of region growing algorithm. In this experiment, the size of radius is 15 pixels in liver segmentation, and 10 pixels in kidney segmentation. Same morphological structuring element is used in the same MR image during the dilation and the erosion operation. The blue contour is close to the real boundary of abdominal organs as can be seen in Figure 5, but boundary leakage is still observable.

**Figure 6:**
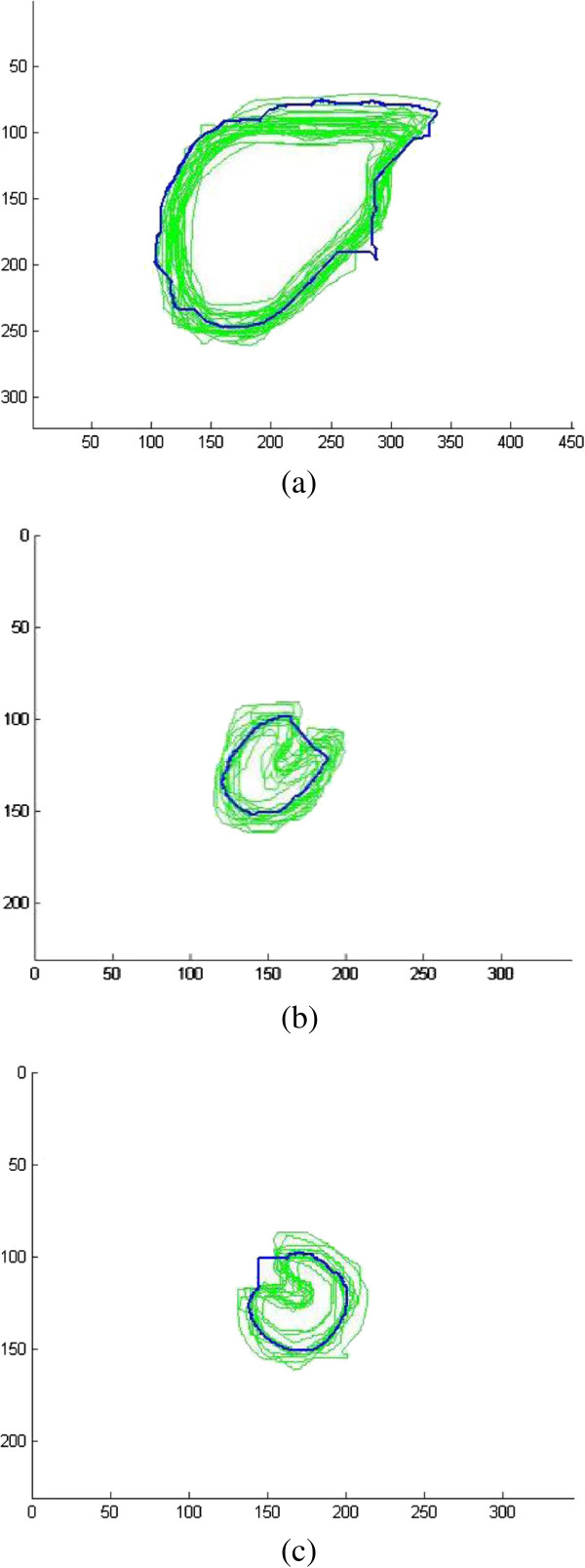
Shape template set and initial contour registration, (a) liver; (b) right kidney; (c) left kidney.

**Figure 7:**
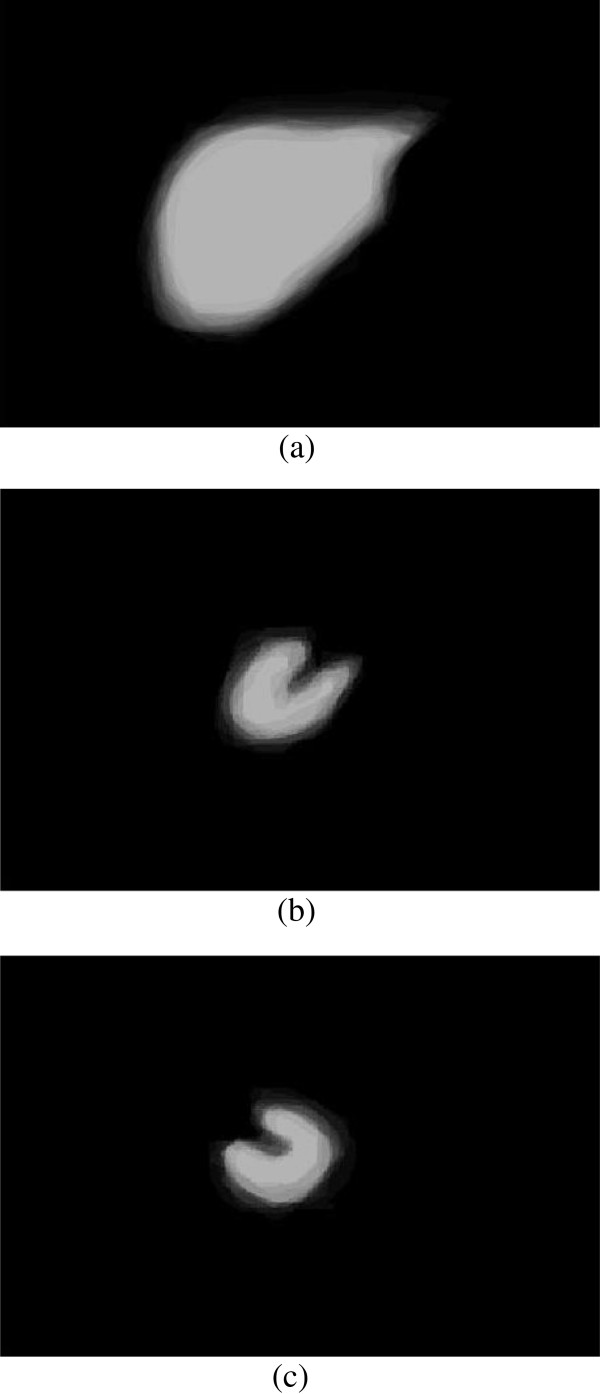
Shape priors, (a) liver; (b) right kidney; (c) left kidney.

**Figure 8:**
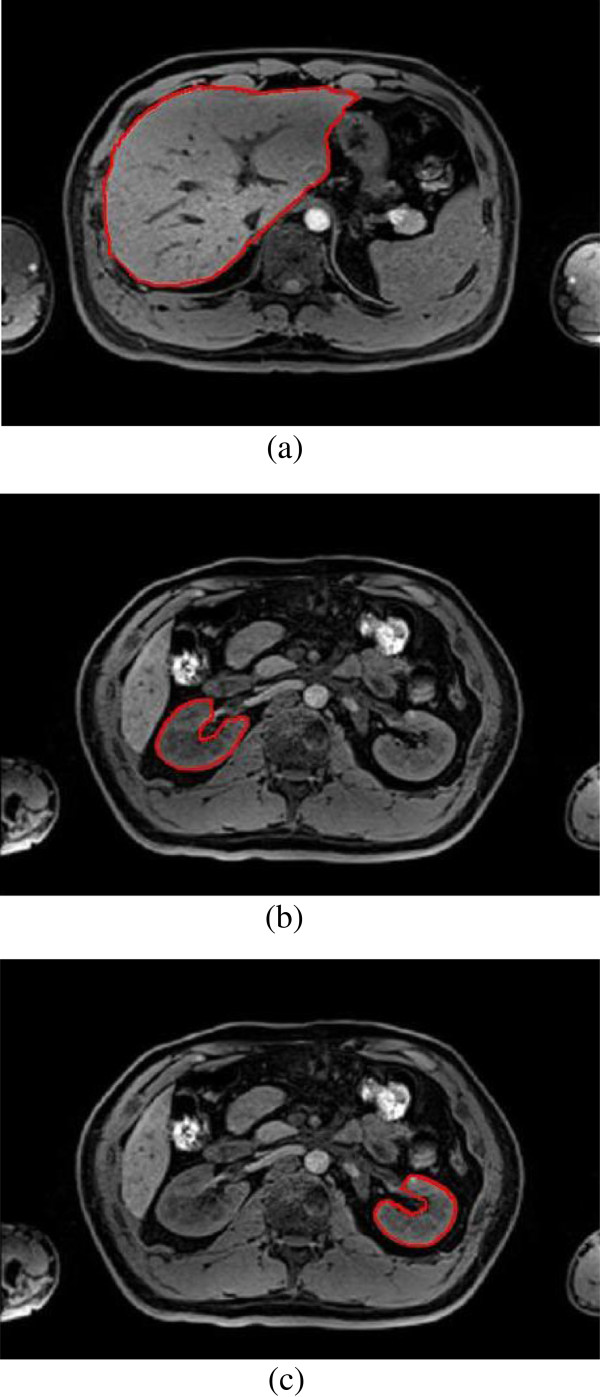
**Segmentation results of proposed novel method, (a) liver (*η*** **=0.65); (b) right kidney (*η*** **=0.72); (c) left kidney (*η*** **=0.75).**

Finally, the shape priors are added into the segmentation to guide the contour more accurate to the real boundary. The size of the training shape template set is 30, and a registration is performed between initial erosion contour (blue contour) and all shape templates (green contour). The result is shown in Figure 6. Figure 7 demonstrates the shape priors which is obtained by training the shape template set using KPCA method. Finally, KGC is combined with shape priors to segment the region inside initial dilation contour (yellow contour) based on function (14). The result of segmentation is shown in Figure 8, from which we can find the boundary leakage is eliminated and the segmentation becomes more correct and accurate.

### Quantitative verification

In order to verify the proposed segmentation method, quantitatively experiments are performed to compare our method and other three methods: DRLSE [11], initial erosion contour, KGC in the initial dilation contour without shape priors. The comparison is based on two quantitative performance measures: the probabilistic rand index (PRI) and the variation of information (VoI) [26-29].

The PRI counts the fraction of pairs of pixels whose labels are consistent between the computed segmentation and the ground truth. The VoI metric defines the distance between two segmentations like the average conditional entropy. Since the result segmentation boundaries of the proposed method are very close to the ground truth, visible difference between them cannot be identified, for this reason the PRI and VoI are used to quantify the segmentation results.

**Figure 9:**
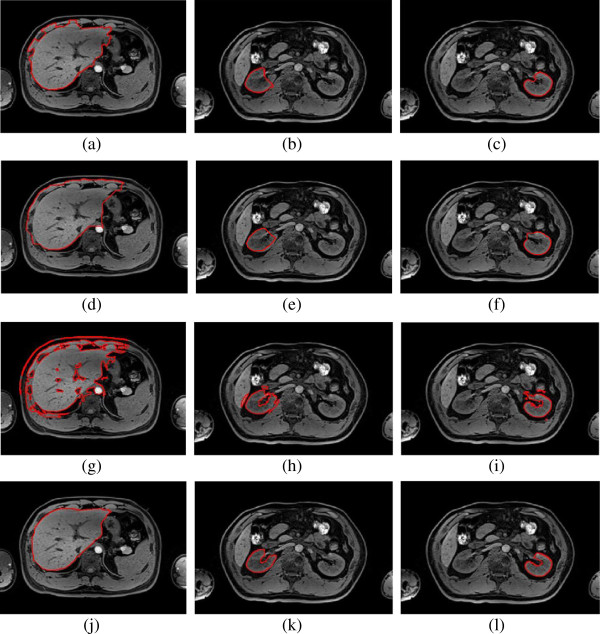
**The results of different algorithms on liver, right kidney and left kidney from left to right of each row in order, (a-c) DRLSE; (d-f) initial erosion contour; (g-i) KGC in the initial dilation contour without shape priors; (j-l)the proposed method (*η*** **=0.65, 0.72, 0.75).**

For each segmentation method, a higher value of PRI and a lower value of VoI imply that the segmentation results are closer to the expert manual segmentation. The statistic data are illustrated in Table 1, and the results of different segmentation algorithms are shown in Figure 9. As can be seen in the Table 1, no matter whether the abdominal organ is liver, right kidney or left kidney, the proposed method has the highest PRI values and the lowest VoI values. As shown in Figure 9, the segmentation with the proposed method has better performance than the other methods. The KGC with shape priors based on KPCA can overcome the boundary leakage and segment every abdominal organ independently without incorrect segmentation of the similar tissues. It indicates that both for liver and for kidney segmentation, the proposed method is better than the other methods.

**Table 1:** The PRI and VI of different methods in liver and kidneys

**Measures**	**PRI**			**VoI**		
	**Liver**	**Right kidney**	**Left kidney**	**Liver**	**Right kidney**	**Left kidney**
DRLSE	0.9808	0.9911	0.9930	1.6648	0.3823	0.3595
Initial erosion contour	0.9155	0.9906	0.9917	1.8772	0.3485	0.3311
KGC in the initial dilation contour without shape priors	0.8903	0.9808	0.9913	2.1212	0.5351	0.3965
The proposed method	0.9912	0.9983	0.9980	1.6193	0.3205	0.3217

### Parameter adjustment

The value of parameter represents the relative influence of KGC data term and shape priors term. In Figure 8, the value of *η* increases gradually from liver to right kidney. Since the incorrect segmentation in the Figure 9(g-i) decreases gradually from liver to right kidney, the KGC data term weighted in the energy function become high. If *η* has a high value, the weight of KGC data term is high too and the weight of shape priors term is small. For different organs different organs, the value of *η* can be adjusted to optimize segmentation performance.

**Figure 10:**
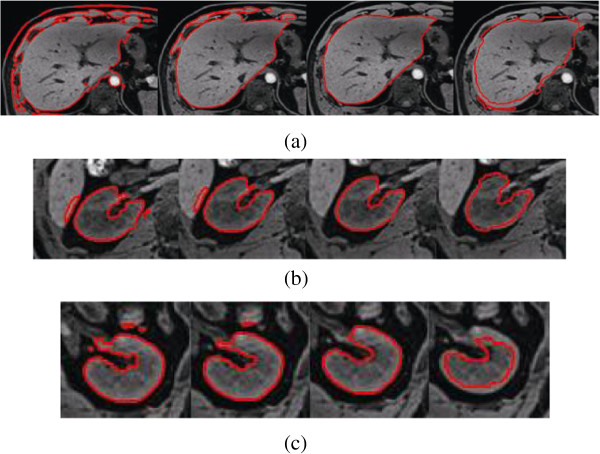
**The segmentation of different *η *** **using the proposed method, from left to right in each row, (a)liver:** ***η*** **= 0.8, 0.7, 0.65, 0.6; (b) right kidney: *η*** **= 0.8, 0.75, 0.72, 0.7; (c) left kidney: *η*** **= 0.9, 0. 8, 0.75, 0.7.**

As shown in Figure 10, when =0.65, 0.72 and 0.75, the segmentation of liver, right kidney and left kidney reach the best accuracy. From the third row of Table 1, the value of PRI is 0.8903, 0.9808 and 0.9913, and the value of VoI is 2.1212, 0.5351 and 0.3965 from liver to right kidney when using KGC inside initial dilation contour without shape priors. In another words, the segmentation error decreases from liver to right kidney, as also shown in Figure 9 (g-i).

As mentioned above, by adjusting the value of parameter *η*, the segmentation can be optimized. If the incorrect segmentation proportion of KGC inside initial dilation contour without shape priors is small, the weight of KGC data term is high, so that the value of *η* become bigger. On the contrary, if the incorrect segmentation proportion of KGC inside initial dilation contour without shape priors is high, the value of *η* should become smaller. In this way, a satisfying segmentation result can be obtained finally.

## Discussion

Because of the noise, weak boundary, intensity inhomogeneity and similar intensities among different abdominal organs, the segmentation of abdominal organs is normally considered a challenging task. Furthermore, the deformation caused by individual difference and respiratory movement makes the segmentation task even more difficult.

**Figure 11:**
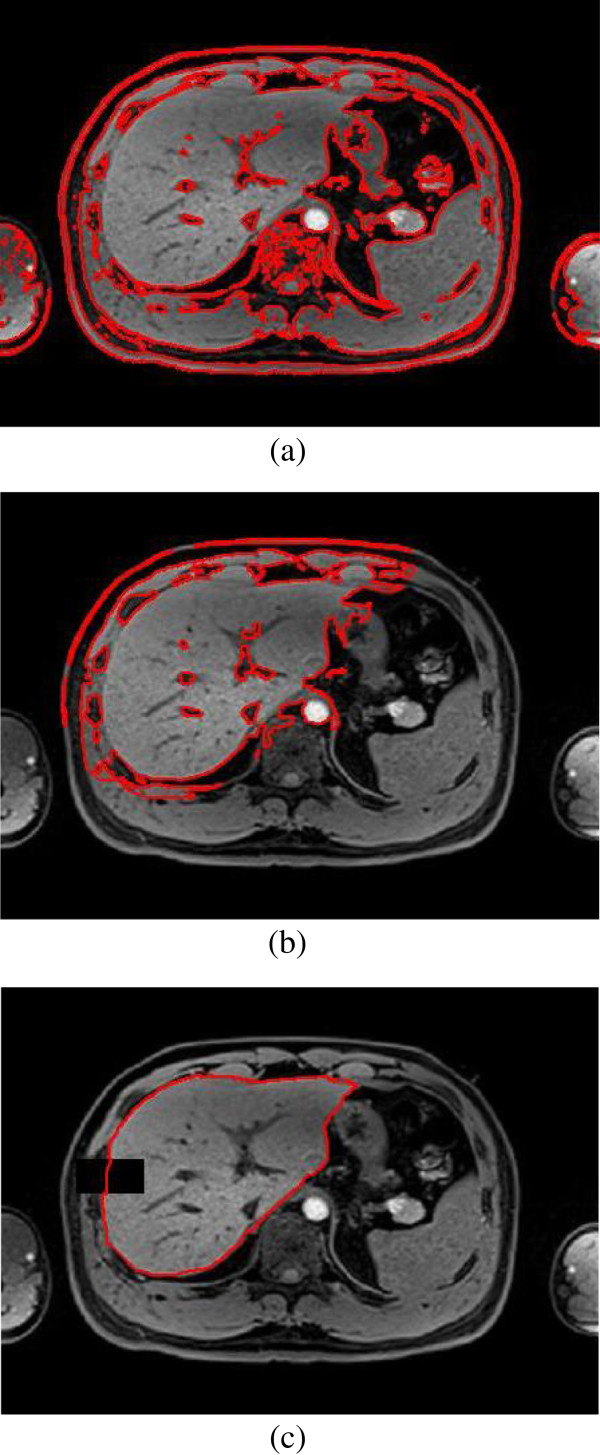
Comparison of different constraint with KGC on liver, (a) KGC’s segmentation for liver without initial contour, (b) KGC in the initial dilation contour without shape priors, (c) the proposed method’s segmentation with shelter.

KGC is a fully automatic segmentation algorithm based on graph cuts. If the KGC algorithm is used alone to segment abdomen MR image, the segmentation result is not satisfying as shown in the Figure 11 (a). The blood vessels in organ and the overlaps among abdominal organs affect the segmentation, thus, a fully complete boundary of liver or other organs cannot be obtained. To make the segmentation procedure focus on a given organ, the initial contour is produced by region growing algorithm and morphology operation. But this is considered not enough. The segmentation result can be seen from Figure 11(b). For this reason some shape priors has been used to make the segmentation algorithm more robust and accurate. Since KPCA can handle nonlinear deformable information, the shape priors based on KPCA is integrated into KGC. Moreover, if a shelter is placed onto the target’s boundary, a satisfying segmentation result can be also obtained. This is validated by experiments as shown in Figure 11 (c).

Additionally, to develop reliable prior knowledge, we should choose patients with similar age and weight. In this way their shape prior would guide to the correct segmentation.

On the clinical side, the work assumed that the geometric inaccuracy, such as distortion, does not exist. Because of this, the MR imaging technology has experienced a rapid development to overcome the magnetic field inhomogeneity. Nowadays, the image spatial resolution can be very high and geometric distortion of MR images can be ignored. However, if a more accurate segmentation is needed, geometric distortion should be corrected at first. This will be investigated in future works.

## Conclusions

In this paper, a novel method is proposed to segment abdominal organs integrating kernel graph cuts with KPCA shape priors after a series of pre-processing on the abdomen MR images. The morphology operation can eliminate the isolated small region after region growing algorithm. The kernel graph cuts is a fully automatic segmentation algorithm, and it also has global minimization and polynomial time complexity characteristics. The shape priors, generated by pre-image projection via KPCA, can handle nonlinear deformation. Experiments on liver and kidney segmentation of abdomen MR image showed that the novel method can obtain satisfying results. The kernel function used in this method is gauss function, and other kernel functions have not been tested yet. Currently, the seed point is obtained manually, but automation will be considered in future works. For the time performance, the algorithm can be parallelized on Graphic processors to achieve higher performance.

## Abbreviations

MR: Magnetic resonance; KGC: Kernel graph cuts; KPCA: Kernel principle component analysis; PRI: Probabilistic rand index; VoI: Variation of information; DRLSE: Distance regularized level set evolution; AAM: Active appearance model; PCA: Principle component analysis; GCBAC: Graph cuts based active contours.

## Competing interests

The authors declare they have no competing interests.

## Authors’ contributions

QL suggested the algorithm for images analyzing and processing, implemented it and analyzed the images. WJ gave the suggestion on algorithm analysis, experiment discussion and manuscript modification. TW, NG, SF and LL performed the acquisition of the abdominal MR images and manuscript discussion. JG and YQ expressed opinions on the evaluation metric of the segmentation results. All authors have read and approved the final manuscript.
